# Autoregulation and Heterogeneity in Expression of Human Cripto-1

**DOI:** 10.1371/journal.pone.0116748

**Published:** 2015-02-06

**Authors:** Pojul Loying, Janvie Manhas, Sudip Sen, Biplab Bose

**Affiliations:** 1 Department of Biotechnology, Indian Institute of Technology Guwahati, Guwahati, India; 2 Department of Biochemistry, All India Institute of Medical Sciences, New Delhi, India; Georgia Regents University, UNITED STATES

## Abstract

Cripto-1 (CR-1) is involved in various processes in embryonic development and cancer. Multiple pathways regulate CR-1 expression. Our present work demonstrates a possible positive feedback circuit where CR-1 induces its own expression. Using U-87 MG cells treated with exogenous CR-1, we show that such induction involves ALK4/SMAD2/3 pathway. Stochasticity in gene expression gives rise to heterogeneity in expression in genetically identical cells. Positive feedback increases such heterogeneity and often gives rise to two subpopulations of cells, having higher and lower expression of a gene. Using flow cytometry, we show that U-87 MG cells have a minuscule subpopulation with detectable expression of CR-1. Induction of CR-1 expression, by exogenous CR-1, increases the size of this CR-1 positive subpopulation. However, even at very high dose, most of the cells remain CR-1 negative. We show that population behavior of CR-1 induction has a signature similar to bimodal expression expected in a transcriptional circuit with positive feedback. We further show that treatment of U-87 MG cells with CR-1 leads to higher expression of drug efflux protein MDR-1 in the CR-1 positive subpopulation, indicating correlated induction of these two proteins. Positive feedback driven heterogeneity in expression of CR-1 may play crucial role in phenotypic diversification of cancer cells.

## Introduction

Expression of genes involved in embryonic development is spatially and temporally regulated through multiple layers of transcriptional control [1]. Aberrations in such controls, in adults, are often associated with development and progression of cancer. Transcriptional control may involve positive and negative feedback [2]. Feedback loops provide precise control over gene expression [3]. Switch-like behavior and oscillation in gene expression also involve feedback loops [4]. Feedback in a transcriptional circuit also affects the cell-to-cell variability or heterogeneity in gene expression [5].

Gene expression is inherently noisy and a population of clonally derived isogenic cells always has heterogeneous expression of any gene [6]. Such heterogeneity plays a crucial role in embryonic development [7] and cancer [8]. The noise in gene expression originates primarily from the stochasticity in the underlying processes. It has now been established that a positive feedback increases heterogeneity in gene expression and may even create two subpopulations having low and high expression [6,9,10]. A positive feedback can give rise to bistability [10]. A bistable system has two stable steady states, with lower and higher expression of the target gene. In such a system, for a given inducing signal, cells can have either higher expression or lower expression of the gene [10]. This gives rise to a mixed population of cells with bimodal distribution in expression [10]. Due to the inherent stochasticity in transcriptional processes, a positive feedback can lead to bimodal gene expression even without bistability [11,12]. Bimodal gene expression, due to positive feedback in transcription, has been observed for genes involved cellular differentiation [13] and embryonic development [14].

Human Cripto-1 (CR-1) is an oncofetal protein. It is essential for signaling by Nodal, a key morphogen in embryonic development [15]. CR-1 is expressed as a membrane-bound molecule and subsequently released in soluble form [15]. Both membrane-bound and soluble CR-1 are functional [15]. Together, Cripto and Nodal control various processes in embryonic development, like formation of primitive streak, establishment of left-right axis and mesendoderm induction [15]. CR-1 is expressed in various human embryonic stem cell lines [16] and in human induced pluripotent stem cells [17]. It is overexpressed in various types of cancers and promotes proliferation of cancer cells, metastasis, and angiogenesis [18].

Multiple pathways control expression of CR-1. It has been shown that NANOG, OCT4, β-catenin, HIF-1α activates CR-1 expression [19–22]. On the other hand, germ cell nuclear factor (GCNF) represses its expression [23]. TGF-β also controls expression of CR-1. It binds to TβRI/TβRII and phosphorylates SMAD2/3 that forms complex with SMAD4. This complex translocates to nucleus and activates expression of target genes by binding to SMAD binding elements (SBEs). CR-1 promoter has multiple SBEs and Mancino *et al*. [24] have shown that treatment with TGF-β1 induces expression of CR-1 in human embryonal and colon cancer cell lines. CR-1, along with Nodal, binds to type I activin receptor ActRIB (ALK4) and activates SMAD2/3 pathway [15]. Therefore, one can postulate that CR-1 would also trigger its own expression through SMAD2/3. Such regulation would create a positive feedback loop: CR-1 expressed in a cell would go to the cell surface and would activate ALK4/SMAD2/3 pathway, thereby inducing further expression of CR-1. Such positive feedback may increase the heterogeneity in expression of CR-1 and may even lead to bimodal distribution of expression of CR-1. In this work we investigate the existence of such an autoregulatory mechanism and explore the heterogeneity in expression of CR-1 in a population of cells.

## Materials and Methods

### Cell culture and recombinant proteins

Human glioblastoma cell line U-87 MG, human breast adenocarcinoma cell line MCF-7 and human colorectal adenocarcinoma cell line HT-29 were procured from the Cell Repository of National Centre for Cell Sciences, Pune, India and maintained in DMEM (Gibco) supplemented with 10% fetal bovine serum (Gibco) and Antibiotic-Antimycotic (Gibco). In all experiments, U-87 MG cells were treated with various recombinant proteins in absence of serum. Recombinant GST-tagged human CR-1 (CR1-GST) and recombinant GST were expressed in *E*. *coli* and purified, as reported earlier [25]. Recombinant human CR-1 expressed in an insect expression system was purchased from R&D Systems. CR-1 cloned in pCI-neo vector [26] was used to overexpress it in stably transfected MCF-7 cells. C-terminal-truncated CR-1 (1^st^—169^th^ amino acid) cloned in pCI-neo vector was used to overexpress CR-1 in soluble form, in stably transfected MCF-7 cells and conditioned media of these cells was used for experiments. Recombinant human TGF-β1 was purchased from Gibco.

### Estimating Gene expression

Total RNA was isolated using TRI-reagent (Sigma) as per manufacture’s protocol. cDNA was synthesized by reverse transcription using RevertAid H Minus Reverse Transcriptase (Thermo Scientific) and random hexamer (Fermentas) as primer. PCRs were performed using BioMix Red (Bioline) on Palm Cycler thermal cycler (Genetix Biotech Asia Pvt. Ltd.). Primers used for PCRs are listed in S1 Table. PCR products were resolved by agarose gel electrophoresis, stained with ethidium bromide and documented using a gel documentation system (Gel Logic 1500, Kodak). Real-time PCRs were performed using SYBR green (Power SYBR Green PCR Master mix, Applied Biosystem) on 7500 Real-Time PCR System (Applied Biosystem). Cyclophilin was used as the endogenous control. Efficiency of each reaction was calculated using LinRegPCR [27]. Fold change in expression of target genes in comparison to the endogenous control was calculated by ΔΔCt method [28].

### Western blot analysis

Cells were lysed using RIPA buffer with PMSF (1 mM), sodium fluoride (50 mM) and sodium orthovanadate (1 mM). Total protein content of each lysate was estimated by Lowry’s method using BSA as a standard. Samples were resolved by SDS-PAGE and transferred to PVDF membrane (Millipore). Blots were probed with appropriate primary and HRP-conjugated secondary antibodies. Antibodies used in different experiments are listed in S2 Table. Blots were developed using chemiluminescence reagents (Super signal West Dura, Thermo Scientific) and imaged using a gel documentation system (Gel Logic 1500, Kodak).

### mRNA stability assay

A method similar to that reported by Hennes *et al*. [29] was used. Cells were treated with CR1-GST (200 ng/ml) or equivalent amount of PBS. After 16 hr of treatment, cells were treated with 5 μg/ml of actinomycin D (Himedia) and incubated for different time points in presence or absence of CR1-GST. Total RNA was isolated at these specific time points. Subsequent to synthesis of cDNA, real-time PCR was used to measure CR-1 transcript as described earlier.

### Flow Cytometry

Cells were detached using enzyme free dissociation buffer (Invitrogen), washed using PBS with 0.1% BSA and incubated in PBS with 2% BSA for 15 min at room temperature. Subsequently, cells were stained for cell surface CR-1 and MDR1 using mouse anti-human Cripto-1-PE (R&D system) and mouse anti-human P Glycoprotein-FITC (Abcam) respectively as per the manufacturers’ protocols. Wherever required, cells were also stained by corresponding isotype control antibodies. Single or double stained cells were analyzed in FACSCalibur flow cytometer (BD Biosciences) and data was collected using CellQuest Pro software (BD Biosciences). CR-1 and MDR1 were detected in FL2-H and FL1-H respectively, both in log mode. For each sample, data of 20,000 cells were collected. Data was analyzed using FCS Express 4 (De Novo Software). Data of each sample was gated first in FSC-SSC dot plot and then in the histogram for FL2-H to remove debris, and extreme data points. In general, more than 97% cells were retained after gating for further analysis. CR-1 positive population was identified by Overton histogram subtraction [30]. Cells treated with highest amount of CR1-GST and stained with isotype control antibody were used as control for subtraction.

For cell sorting, U-87 MG cells were treated with CR1-GST (400 ng/ml) for 24 hr and stained with mouse anti-human Cripto-1-PE mAb or Isotype control antibody as mentioned above. CR-1 positive and CR-1 negative populations were sorted out using FACSAria III (BD Biosciences) equipped with BD FACSDiva 6.0 software. Post sorting, total RNA was isolated using TRI reagent.

### Simulation of noise behavior

We simulated a mixed population of 20,000 cells. It had two subpopulations. One subpopulation is called “Low cells” and the other is called “High cells”. Each member of this population had an assigned random number that represents expression level of a protein in that cell (in arbitrary unit). This value was taken from a log-normal distribution, with specific mean and variance. The mean and variance for Low cells was kept constant at 5 and 50 respectively. The mean for High cells was varied from 10 to 100. The variance for this subpopulation was varied, so that γ = variance/mean = 10, 100, and 1000. Relative size of these two subpopulations was varied from 0 to 100%. Simulation for each parameter combination was repeated 1000 time and average mean and CV of the whole population were calculated. Simulations were performed using MATLAB R2013b (MathWorks).

### Data analysis

Two-way ANOVA or one-way ANOVA was used depending upon the type of experiment. Mann-Whitney Rank Sum Test was used for data derived from real-time PCR experiments. SigmaPlot was used to generate graphs, for data fitting and statistical analysis. Means of multiple data points were plotted, as mentioned in figure legends. Error bars represent standard deviations.

## Results

### Cripto-1 induces its own expression

**Fig 1:**
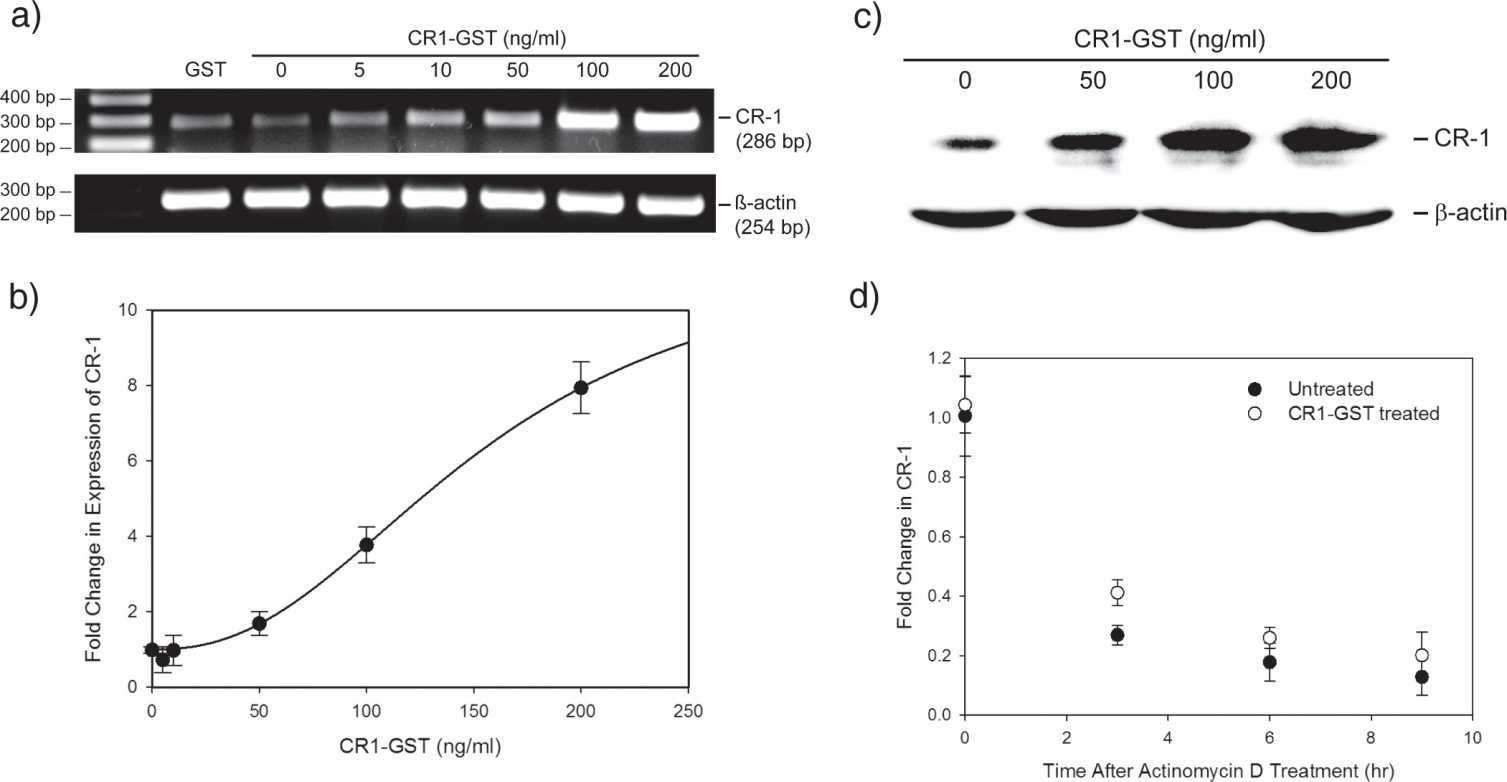
Treatment with recombinant CR-1 induces CR-1 expression. U-87 MG cells were treated with different doses of CR1-GST or GST (200 ng/ml) for 24 hr. Expression of CR-1 was measured by (a) RT-PCR and (b) real-time PCR. Data of real-time PCR was fitted to Hill equation (fold change = 1+ 10.85 × dose^2.37^/(156.99^2.37^+ dose^2.37^); R^2^ = 0.98). (c) Western blot to detect induction of CR-1 in U-87 MG cells treated with different doses of recombinant CR-1. (d) Fold change in CR-1transcript at different time points after treatment with actinomycin D in presence and absence of CR1-GST. No significant difference between treated and untreated cells (Rank Sum Test, p = 0.127). In (b) and (d) each data point represents average of four independent experiments.

U-87 MG cells express molecules involved in Nodal/ALK4/SMAD2/3 pathway (S1 Fig.). We have treated these cells with recombinant GST-tagged CR-1 (CR1-GST) and expression of CR-1 in these cells was measured by RT-PCR. Expression of CR-1 in U-87 MG cells is low and treatment with exogenous CR-1 increases its expression in these cells in a dose dependent fashion (Fig. 1A). The induction was confirmed by real-time PCR (Fig. 1B). Such induction was also observed when U-87 MG cells were treated with recombinant CR-1 expressed in mammalian and insect expression systems (S2 Fig.).

Inducible gene expression systems often have sigmoidal behavior represented by Hill function [31]. The data shown in Fig. 1B is sigmoidal in nature and fits with Hill function having Hill coefficient 2.37. A Hill coefficient > 1 indicates cooperativity in the transcriptional circuit [31].

Induction of CR-1 expression by exogenous CR-1 was confirmed at protein level by Western Blot. As shown in Fig. 1C, treatment with recombinant CR-1 increases the amount of endogenous CR-1 in a dose dependent fashion. Concurrent increase in the level of CR-1 transcript and protein, can be achieved by two mechanisms: increase in the transcription of the gene and by increasing the stability of its mRNA. We performed mRNA stability assay using actinomycin D to check the effect of exogenous CR-1 on the stability of CR-1 mRNA. As shown in Fig. 1D, subsequent to actinomycin D treatment, amount of CR-1 transcript, in untreated U-87 MG cells, reduced in a time dependent fashion typical to the first order degradation of mRNA. The decay of CR-1 mRNA was not significantly different in CR-1 treated cells. This indicates that treatment with CR-1 does not affect the stability of CR-1 mRNA. Based on these experiments we concluded that treatment with CR-1 induces transcription of CR-1 in U-87 MG cells.

**Fig 2:**
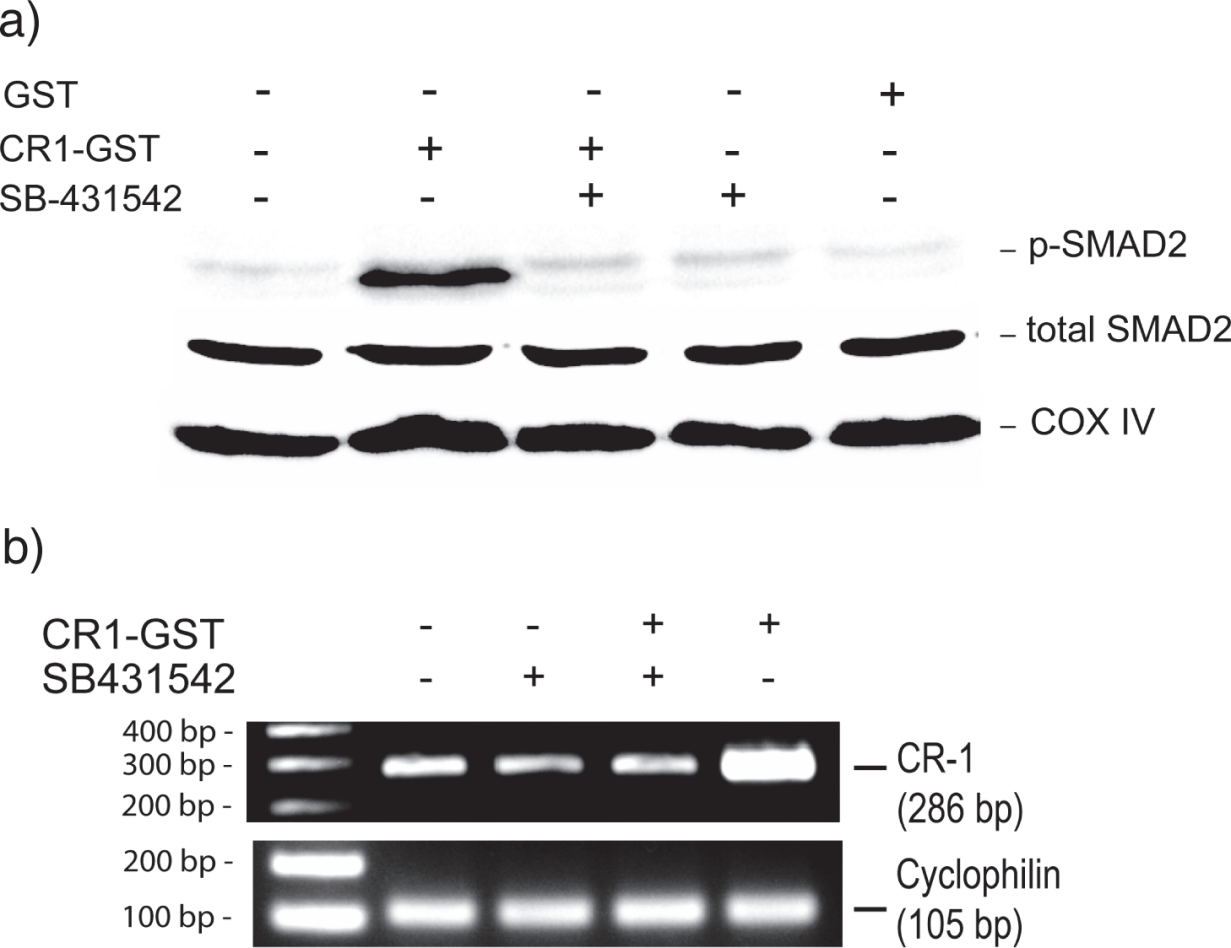
Treatment with CR-1 induces expression of CR-1 through ALK4/SMAD2/3 pathway. (a) Western blot to detect phosphorylation of SMAD2 in U-87 MG cells treated with different combinations of CR1-GST, GST and ALK4 inhibitor (SB-431542) for 15 min. (b) RT-PCR to check expression of CR-1 after 24 hr treatment of U-87 MG cells with different combinations of CR1-GST and SB-431542.

Subsequently we have investigated the involvement of ALK4/SMAD2/3 pathway in CR-1 mediated induction of CR-1 expression. We have treated U-87 MG cells with recombinant CR-1 in presence and absence of an ALK4 inhibitor (SB-431542). As shown in Fig. 2A, treatment with CR-1 activates SMAD2/3 pathway with increase in phopsho-SMAD2. ALK4 inhibitor blocks such activation (Fig. 2A). Inhibition of ALK4 also blocks CR-1 mediated induction of CR-1 expression (Fig. 2B). We have treated two other cell lines, MCF-7 and HT-29 with recombinant CR-1. SMAD4 is expressed in MCF-7. However, HT-29 is a SMAD4-deficient cell line [32,33]. Expression of CR-1 was not detected in untreated MCF-7 cells. However, treatment with recombinant CR-1 induced expression of CR-1 in these cells (S3 Fig.). HT-29 cells have low expression of CR-1 and treatment with recombinant CR-1 failed to increase expression of CR-1 in these cells (S3 Fig.). These results confirm that CR-1 activates ALK4/SMAD2/3 pathway and that leads to induction of transcription of CR-1.

### Heterogeneity in induction of CR-1 expression

Mathematical and experimental works have shown that positive feedback loop in a transcriptional circuit can lead to bimodality in gene expression [9–11]. In such cases, induction of gene expression in a population of clonally identical cells would lead to formation of two subpopulations having higher and lower expression of the gene.

Treatment with recombinant CR-1 induces expression of CR-1 through Alk-4/SMAD2/3 pathway. This would increase endogenous CR-1 on the cell surface. These cell surface CR-1 molecules will also induce the same pathway, thereby creating a positive feedback. Therefore, one can expect that induction of this autoregulatory pathway of CR-1 may lead to the emergence of two subpopulations.

We have used flow cytometry to investigate the heterogeneity in induction of CR-1 expression in U-87 MG. Staining for CR-1 in untreated U-87 MG cells was very weak and it was not possible to visually differentiate between the fluorescence histograms of cells stained with anti-CR1 antibody and isotype control antibody. This indicates either these cells had very low expression of CR-1 or the antibody used in our experiment was not functional. Therefore, we used a stably transfected MCF-7 cell line overexpressing full-length CR-1 as a positive-control. We observed that the anti-CR1 antibody used in our work is functional and can clearly differentiate cells overexpressing CR-1 from cells transfected with empty vector (S4 Fig.).

As the expression of CR-1 was low and the background reading was high, we used histogram subtraction to identify CR-1 positive cells. In this method, flow cytometry readings for the cells stained with isotype control antibody were subtracted from that of cells stained with anti-CR-1 antibody using Overton histogram subtraction method [30]. This allowed us to estimate percentage of cells that have detectable level of CR-1 expression. We call these cells CR-1 positive. Rest of the cells having non-detectable level of CR-1 expression were called CR-1 negative. We found that only an extremely minor population (~7%) of U-87 MG cells were CR-1 positive.

**Fig 3:**
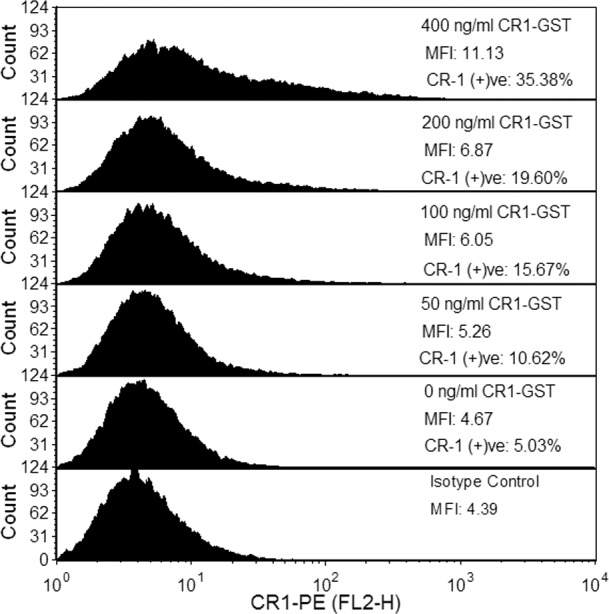
Flow cytometry to detect heterogeneity in expression of CR-1. U-87 MG cells were treated with different doses of CR1-GST for 24 hr and expression of CR-1 was measured. Cells treated with CR1-GST (400 ng/ml) and stained with isotype control antibody was used as negative control. This experiment has been repeated and one representative data is shown here.

**Fig 4:**
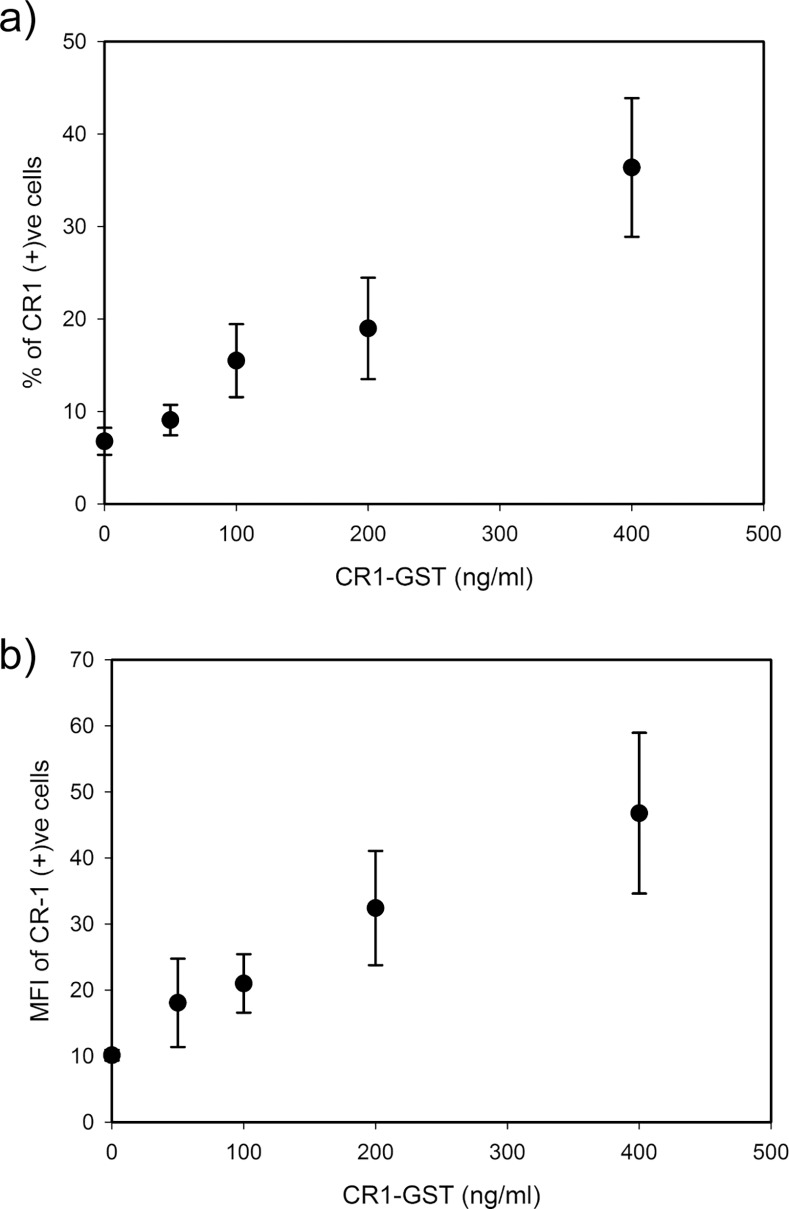
Dose dependent behavior of CR-1 positive subpopulation. U-87 MG cells were treated with different doses of CR1-GST for 24 hr and expression of CR-1 was measured by flow cytometry. (a) Percentage of cells in CR-1 positive subpopulation and (b) level of CR-1 expression, measured in terms of MFI, in cells of CR-1 positive subpopulation. Each data point represents mean of three independent experiments.

Subsequently, U-87 MG cells were treated with different doses of recombinant CR-1 and expression of CR-1 was measured by flow cytometry. Uniform induction of CR-1 expression, in all cells, would move the whole fluorescence histogram for CR-1 towards right-hand side (i.e. to higher values) without changing the shape of the histogram. However, such shift was not observed in our experiment (Fig. 3). We observed that treatment with CR-1 did not induce CR-1 expression in all cells (Fig. 3). Even at the highest dose (400 ng/ml), most of the cells did not have detectable level of CR-1. Only a minority subpopulation was CR-1 positive with detectable level of CR-1 expression. With increase in dose of the recombinant CR-1, the size of the CR-1 positive subpopulation increases fattening the tail of the fluorescence histogram for CR-1 (Figs. 3 and 4A). This indicates that depending on the strength of induction, cells diverge into two groups, one with higher expression of CR-1 and the rest with lower expression. At the same time, level of expression in those CR-1 positive cells also increases (Fig. 4B). Such population behavior has been earlier observed in synthetic transcriptional circuits with positive feedback [9].

Existence of two subpopulations may lead to bimodal distribution in flow cytometry data. However, we have not observed clear bimodal distribution with two distinct peaks in our flow cytometry data. Bimodality in a population is not clearly visible when both the subpopulations are very close to each other [34], as it happened in our experiments. In our experiments, bimodality is further obscured as the background reading (measured in terms of the isotype control) is very high and has a long tail. We looked into the noise in the flow cytometry data to identify a signature of bimodality. Noise in gene expression is usually measured in terms of coefficient of variation (CV), which is the ratio of standard deviation to mean [5]. We calculated mean and CV of FL2-H (i.e. CR1-PE) data in different treatment groups.

**Fig 5:**
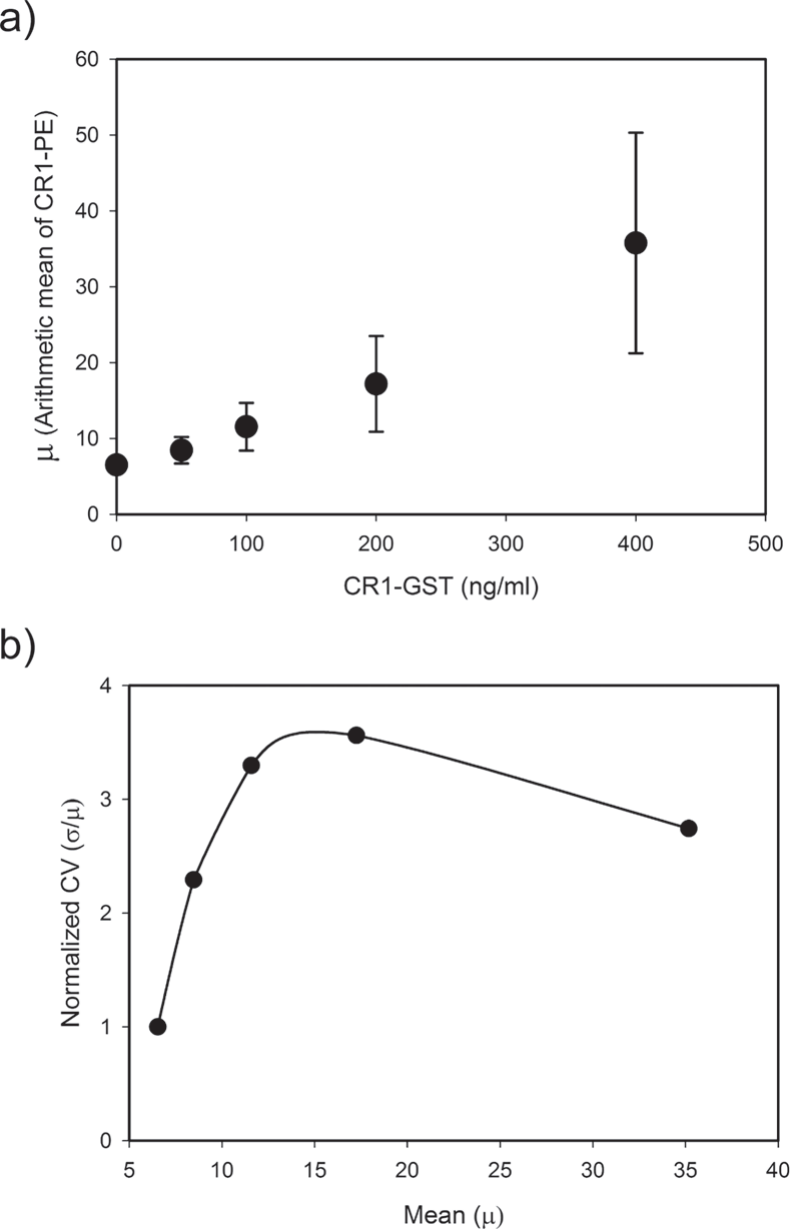
Noise in CR-1 induced expression of CR-1 in U-87 MG cells. Expression of CR-1 was measured by flow cytometry. Panel (a) shows the change in mean of CR1-PE (FL2-H) reading for the whole population of cells with dose of recombinant CR-1. Panel (b) shows the relation between mean and noise in CR-1 expression. Noise is represented in terms of normalized CV. CV of CR1-PE (FL2-H) of each treated sample was normalized by dividing with that of untreated cells. Average results of three independent experiments are shown here.

In our experiments, treatment with CR-1 increased mRNA of CR-1 without changing its stability. That means induction by exogenous CR-1 increases the rate of transcription of CR-1. Distribution of a protein in a population of cells usually follows unimodal distribution. In such systems, increase in the rate of transcription increases mean level of the protein without changing the noise (CV) [35]. In our experiments, mean expression of CR-1 increased with the dose of recombinant CR-1 (Fig. 5A). However, the noise in CR-1 expression was not constant but changed non-monotonically with increase in expression of CR-1 (Fig. 5B). Initially, noise rises with increase in the inducing signal and reaches a maximum. Further increase in dose of recombinant CR-1 leads to increase in mean expression of CR-1 but decrease in noise.

This type of noise pattern can arise due to bimodal population distribution and have been observed earlier [36,37]. In a mixed cell population, mean and CV are decided by the relative sizes and the statistical parameters of each subpopulation. In untreated cells, CR-1 positive subpopulation was minuscule. Statistical parameters of the whole population were decided primarily by the CR-1 negative subpopulation. With increase in the inducing signal, size of the CR-1 positive subpopulation increased and it affected the statistics of the whole population. This population had higher mean and variance. This led to increase in CV of the whole population. After a threshold, effect of the CR-1 positive subpopulation became dominant. With further increase in this population, the CV of the whole population decreased and moved towards that of the CR-1 positive population.

We then simulated a mixed population of cells with two subpopulations, one with a higher mean and variance than the other. Sizes of these subpopulations were varied and simulations were performed to show the effect of relative population sizes on the mean and CV of the whole population. We observed that in certain parameter regimes, the CV of the whole population behaves similar to our observation (S5 Fig.). This confirmed that existence of two subpopulations, with higher and lower expression, could give rise to the noise behavior observed in Fig. 5B.

Cell size can affect the variability in the flow cytometry data. Therefore, we checked the correlation between FSC (an approximate measure of cell size) and FL2-H (measurement of CR-1) in cells treated with different doses of recombinant CR-1. We have not observed any such correlation in our data (S6 Fig.).

**Fig 6:**
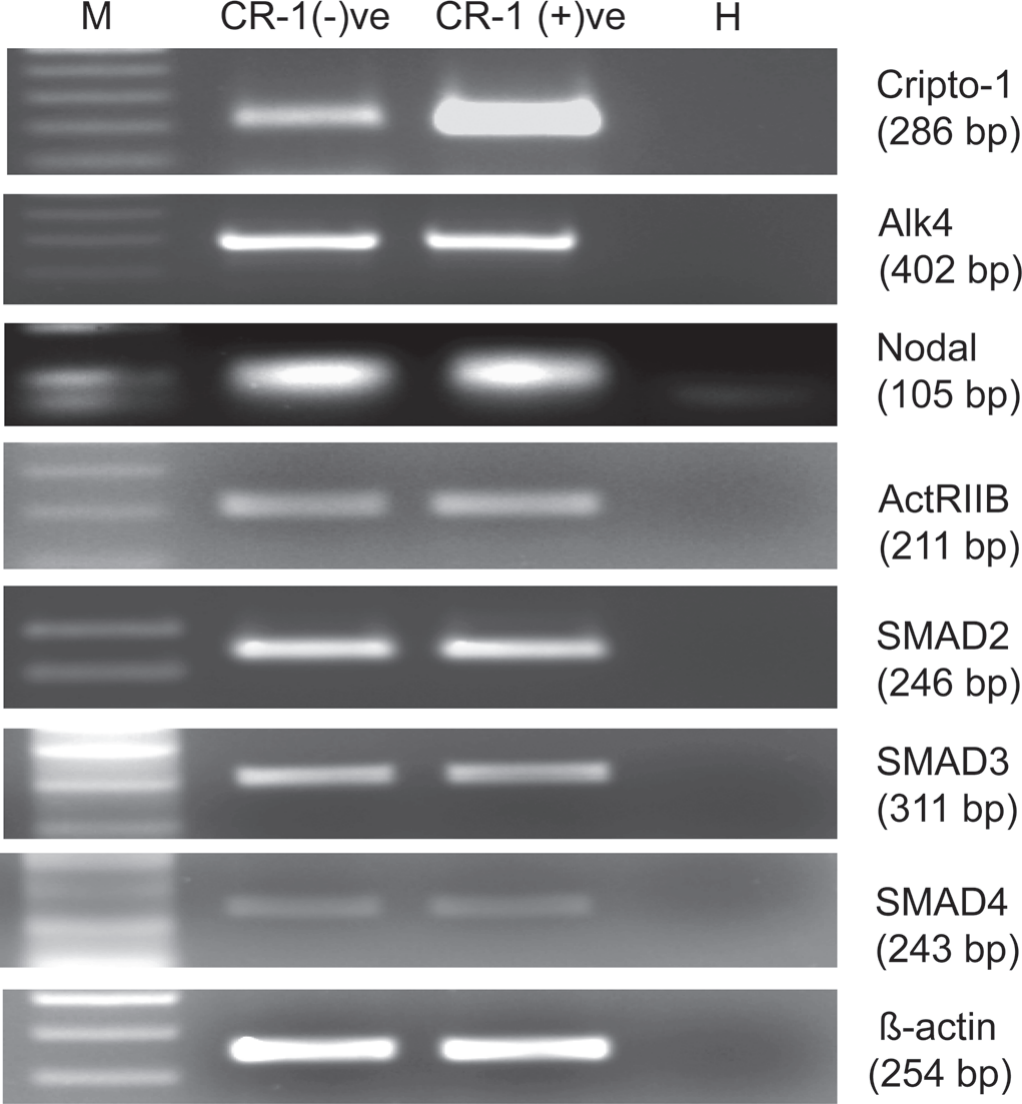
Expression of different pathway molecules in CR-1 positive and negative subpopulations. U-87 MG cells treated with CR1-GST (400 ng/ml) for 24 hr were sorted in two subpopulations and RT-PCR was used to measure gene expression. M: DNA marker, H: Water.

We have argued that stochasticity, with positive feedback, is driving the heterogeneity in CR-1 induction. However, it could also be argued that the majority of U-87 MG cells did not have the machinery required for induction of CR-1 through the ALK4/SMAD2/3 pathway. Therefore, treatment with recombinant CR-1 failed to induce CR-1 expression in those cells. We have treated U-87 MG cells with recombinant CR-1 (400 ng/ml) and subsequently sorted out CR-1 positive and CR-1 negative cells by FACS. RNA was isolated from both the subpopulations and expression of molecules involved in ALK4/SMAD2/3 pathway was checked by RT-PCR. As shown in Fig. 6, these molecules were expressed at similar levels in both the subpopulations. This confirmed that two subpopulations in CR-1-treated cells had not originated due to differences in expression of pathway molecules between two groups of cells. The size of the CR-1 positive population increases with treatment dose. This observation also negates the possibility of emergence of two subpopulations due to existence of two groups of cells having high and low expression of pathway molecules.

### CR-1 co-induces expression of CR-1 and MDR1

**Fig 7:**
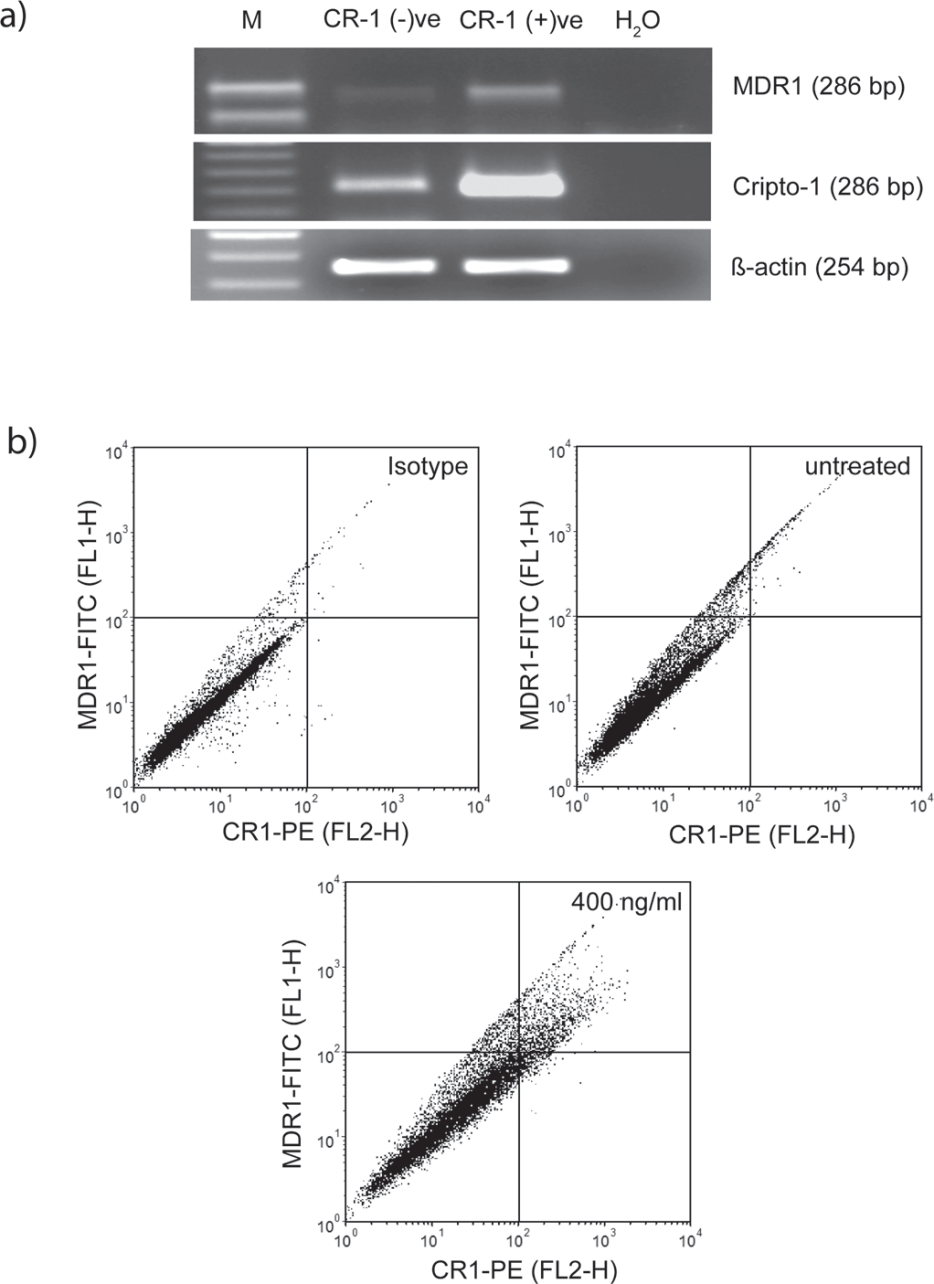
Co-induction of MDR-1 and CR-1. a) U-87 MG cells treated with CR1-GST (400 ng/ml) for 24 hr were sorted in two subpopulations and RT-PCR was used to measure gene expression. b) Cells were treated with CR1-GST (400 ng/ml) or left untreated for 24 hr and expression of CR-1 and MDR1 was measured by flow cytometry. Cells in the right-upper quadrant are positive for both CR-1 and MDR1. Cells treated with CR1-GST (400 ng/ml) but stained with isotype control antibodies was used as negative control.

Strizzi *et al*. [38] have earlier reported that CR-1-expressing melanoma cells have higher expression of the multidrug resistance protein MDR1. We have treated U-87 MG cells with recombinant CR-1 and sorted CR-1 positive and negative subpopulations. Expression of MDR1 was checked in these two subpopulations. We observed that expression of MDR1 is higher in CR-1-positive cells (Fig. 7A). Subsequently, heterogeneity in CR-1 and MDR1 expression was investigated using flow cytometry. We observed that, like CR-1, MDR1 is expressed at detectable level only in a minority subpopulation (~13%) of U-87 MG cells. Treatment with CR-1 (400 ng/ml) increased MDR1 positive population to ~37%. As shown in Fig. 7B, most of these induced cells are positive for both CR-1 and MDR1. Therefore, CR-1 co-induces expression of CR-1 and MDR1 in a minority subpopulation of U-87 MG cells.

**Fig 8:**
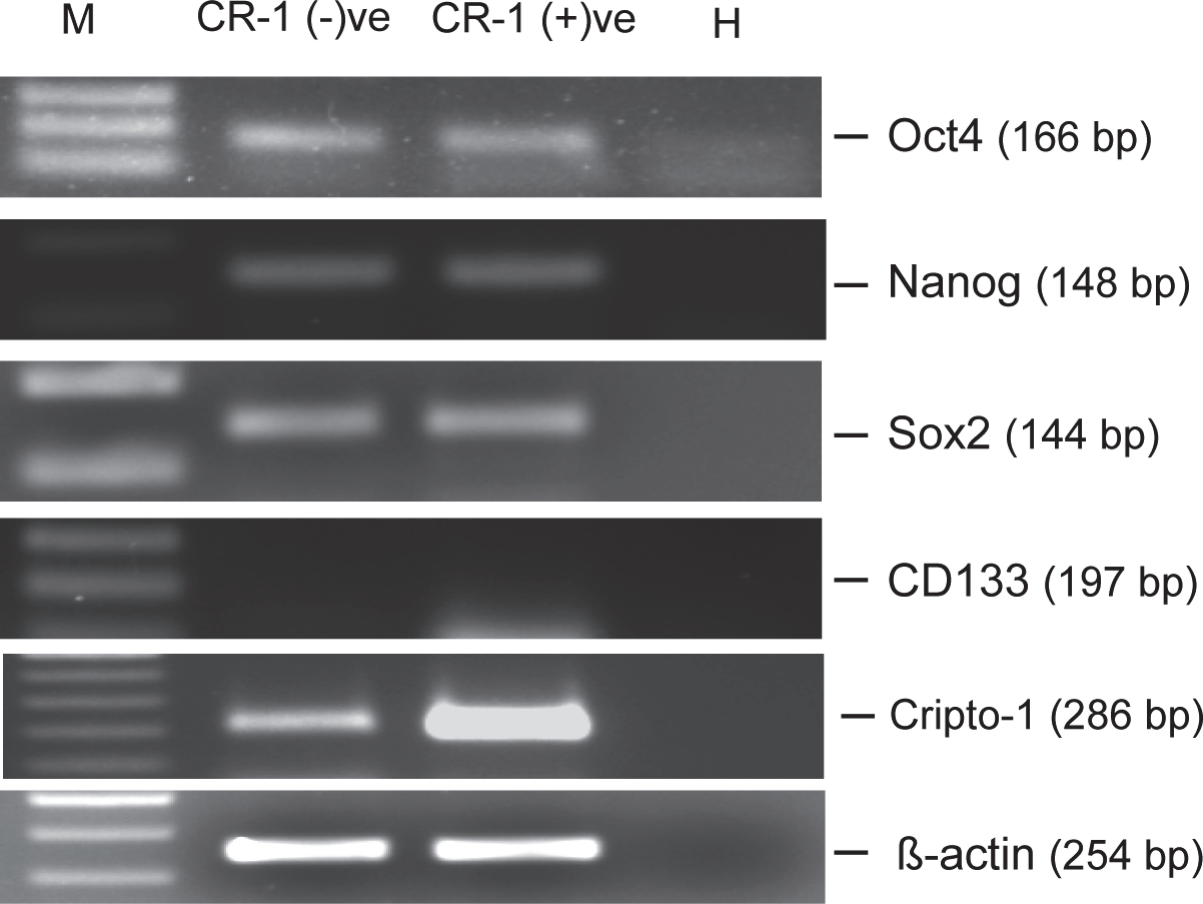
Expression of stemness-related molecules. U-87 MG cells treated with CR1-GST (400 ng/ml) for 24 hr were sorted in two subpopulations and RT-PCR was used to measure gene expression. M: DNA marker, H: Water.

Nakai *et al*. [39] established a cell line derived from spheroid culture of U-87 MG cells that showed drug resistance and higher expression of MDR1. These cells were positive for CD133, a marker for cancer stem cells. Similarly, Strizzi *et al*. [38] had also observed higher expression of OCT4, a marker for stemness, in CR-1 positive melanoma cells. Therefore, we looked into the expression of several markers of cancer stem cells (Fig. 8). We have not observed any such increase in OCT4 in CR-1 positive U-87 MG cells (Fig. 8). U-87 MG cells do not express CD133 [39,40,41] and we have not detected expression of CD133 in both the subpopulations. Further, we observed that NANOG and Sox2, two other markers of stem cells, were expressed at same level in both the subpopulations (Fig. 8).

## Discussion

In this work, we have shown that CR-1 can induce its own expression through ALK4/SMAD2/3 pathway and such induction leads to cellular heterogeneity with only a minority subpopulation having high expression of CR-1.

Cripto-1 is involved in various processes in embryonic development and its aberrant expression is associated with several types of cancer. Being a coreceptor of a morphogen, Nodal, expression of CR-1 requires precise control. TGF-β induces expression of CR-1 through SMAD2/3 pathway [24] and CR-1 is known to activate the same pathway[15]. Based on this information, we hypothesized that CR-1 would induce its own expression through ALK4/SMAD2/3 pathway. To check this hypothesis, we treated human glioblastoma cell line, U-87 MG with recombinant human Cripto-1. Confirming our hypothesis, treatment with recombinant CR-1 induced expression of endogenous CR-1 in these cells, through the ALK4/SMAD2/3 pathway, in a dose dependent fashion.

At transcript level, the dose dependency showed a typical sigmoidal behavior as observed in many inducible systems. We fitted the data to the Hill function and the Hill coefficient was found to be 2.37. In an inducible transcriptional circuit, a Hill coefficient > 1 indicates cooperativity [31]. Transcription in mammalian systems involves multiple transcription factors interacting with each other. Even the same transcription factor may bind to multiple sites in the same promoter and interact with each other. Such interactions lead to cooperativity in transcriptional circuits. SMADs are known to have such cooperativity [42,43]. The promoter region of CR-1 has multiple SMAD-binding elements [24] and one can expect binding of multiple, interacting SMAD complexes at those sites.

Cooperativity in transcriptional control plays a role in cell-to-cell variability in gene expression. Stochasticity in gene expression leads to cell-to-cell variability in gene expression. Such variation gives rise to unimodal distribution of a protein in a population of cells. Graded increase in the inducing signal, usually, generates graded increase in the expression of the protein, uniformly in all cells in a population. Therefore, with increase in inducing signal, the mean level of expression increases, but the population distribution remains unimodal. However, cooperative binding of transcription factors along with positive feedback can give rise to bistability leading to bimodal expression of a gene [44]. In such a system, graded increase in inducing signal would generate a binary response leading to formation of two subpopulations of cells, having higher and lower expression of the gene. Interestingly, noise driven bimodality can arise in a system with positive feedback even without cooperativity and bistability [11].

Induction of CR-1 expression in U-87 MG cells, by exogenous CR-1, indicates existence of a potential autoregulatory positive feedback loop. Additionally, such induction has sigmoidal behavior. Based on these observations, we assumed that activation of this autoregulatory pathway would trigger a binary response. Indeed, our flow cytometry data showed that induction of CR-1 expression by recombinant CR-1 is heterogeneous and creates two subpopulations. The larger subpopulation does not have detectable level of expression of CR-1. We call this CR-1 negative. However, this does not mean that these cells do not express CR-1 at all. RT-PCR with RNA isolated from this subpopulation showed presence of CR-1 transcript, albeit at low, basal level. The other subpopulation, called CR-1 positive, shows expression of CR-1 at higher and detectable level. RT-PCR also confirmed higher level of transcription in these cells.

Interestingly, CR-1 positive population, though minuscule in size, is present even in uninduced cells. With increase in inducing signal, the size of this population increased. This strengthens our claim for the autoregulatory positive feedback in CR-1 expression. There is a basal level of expression of CR-1 in these cells, even in absence of externally added CR-1. The endogenous CR-1 would be present on cell surface and would activate the Nodal/ALK4/SMAD2/3 pathway, creating the positive feedback. Due to this positive feedback and inherent stochasticity, two subpopulations, with lower and higher expression, would emerge. Cells transit between these two populations (or states) probabilistically and the probability of such transition would depend upon the level of induction in each cell. In absence of any external perturbation, these two populations would be in equilibrium. Treatment with external CR-1 would increase the strength of induction thereby increasing the probability of transition from low expressing to high expressing state. This leads to increase in the size of CR-1 positive population.

We have observed another interesting feature in this work. We have observed that treatment with CR-1 co-induced expression of CR-1 and MDR-1 in a subpopulation of U-87 MG cells. MDR1 is a member of ABC transporter family of membrane pumps that use ATP hydrolysis to efflux various drugs from a cell. Hu *et al*. [45] have shown that Doxorubicin selected drug-resistant leukemia cell line has higher expression of CR-1 and MDR1. There exists similarity in transcriptional control of expression of CR-1 and MDR1. Like CR-1, MDR1 is also a target of HIF [46] and β-catenin [47]. TGF-β also induces expression of MDR1 [48] and crosstalk exists between TGF-β/SMAD2/3 and β-catenin pathways [49]. Further, AP-1 also controls expression of MDR1 [50] and it is also known that SMAD interacts with AP-1 [51]. Interestingly, Hu *et al*. [45] have shown that treatment of a multidrug resistant leukemia cell line with an anti-CR-1 antibody led to slight decrease in expression of MDR-1. Therefore, one can speculate that induction in MDR1 expression in U-87 MG cells by CR-1 may be happening through crosstalk between transcription factors controlling MDR1 expression and ALK4/SMAD2/3 pathway. Such crosstalk would lead to correlated population dynamics of expression of CR-1 and MDR-1, as we have observed.

Strizzi *et al*. [38] have shown that cells having higher expression of CR-1, in a heterogeneous melanoma cell population, have stem cell-like characteristics with higher expression of Oct-4. Nakai *et al* [39] had reported that U-87 MG cells having higher expression of MDR1 has higher expression of CD133, a marker of GMB stem cells. We have checked the expression of stem cell markers CD133, Oct-4, NANOG, Sox2 in CR-1 positive and negative subpopulations of CR-1 treated U-87 MG cells. We have not observed any increase in expression of these molecules in CR-1 positive subpopulation. These observations make us believe that the CR-1 positive population in U-87 MG cells may not have stem cell like characteristics. Existence of the miniscule subpopulation of cells having higher expression of CR-1 in melanoma cells as observed by Strizzi *et al*. can also be explained in terms of the positive feedback proposed in our work. We believe CR-1 positive subpopulation emerges spontaneously due to the positive feedback in its transcriptional control, even in absence of expression of other stemness-related molecules. Simultaneous but independent activation of other molecular events may lead to expression of key molecules of embryonic stem cells like NANOG and Oct-4. Some of these molecules further activate expression of CR-1. Additionally, many of those molecules may show heterogeneous expression. Coupling of all these processes may lead to emergence of two subpopulations of CR-1 expressing cell: one with higher expression of CR-1, MDR-1 and other markers of stemness and the other with lower expression of these molecules. Though small in size, such CR-1 high subpopulation may eventually play crucial role in progression of cancer.

Involvement of CR-1 in embryonic pattern formation and progression of cancers is well documented. Here in this work, we have established that induction of CR-1, through the autoregulatory pathway, leads to formation of two subpopulations, with higher and lower expression in CR-1. Spontaneous emergence of such heterogeneity would affect the morphogenic signaling by Nodal and may modulate pattern formation during embryonic development. Non-genetic heterogeneity in gene expression is also observed in cancer cells and such heterogeneity is believed to be involved in emergence of stem cell like cells and drug resistant cells in a tumor. Co-induction of MDR1 and CR-1 in a minority subpopulation of cells strengthens this idea. We know that stochasticity drives heterogeneity in gene expression and the architecture of transcriptional circuits controls and modulates it. This generalized concept was developed, mostly through mathematical analysis and validated experimentally using synthetic gene regulatory circuits designed to test this concept. However, application of this concept to explain existing biological systems is challenging, as such systems are not isolated and are not optimized for testing the hypothesis. This work shows that even then, one can predictively use such a generalized concept to identify and characterize a molecular circuit.

## Supporting Information

S1 Fig Expression of different molecules of Nodal/ALK4/SMAD2/3 pathway in U-87 MG cells. Reverse transcription followed by PCR was used to detect expression of different pathway molecules in U-87 MG cells. C: cDNA, R: RNA, H: water, M: DNA marker.
(TIF)

S2 Fig RT-PCR to check induction of CR-1 expression by two different recombinant CR-1. a) U-87 MG cells were treated with recombinant CR-1 (R&D systems) expressed using an insect expression system. b) U-87 MG cells were treated with two dilutions (1:40 and 1:20) of conditioned media of MCF-7 cells overexpressing soluble CR-1 (C) or having transfected with the vector only (V). In both the experiments cells were treated for 24 hr.
(TIF)

S3 Fig Induction of CR-1 expression in other cell lines. MCF-7, HT-29 and U-87 MG cells were treated with recombinant CR-1 for 24 hr and expression of CR-1 was checked by RT-PCR.
(TIF)

S4 Fig Flow cytometry to check activity of the anti-human CR-1 PE conjugated antibody. MCF CR1: MCF-7 cells overexpressing full-length CR-1 and stained with the anti-CR-1 antibody PE conjugate; MCF7 vector: MCF-7 cells transfected with empty vector and stained with the anti-CR-1 antibody PE conjugate; Isotype control: MCF-7 cells overexpressing full-length CR-1 and stained with isotype control antibody. All cells were stably transfected. MCF-7 cells overexpressing CR-1 were not monoclonal. Rather, transfected clones selected for drug resistance were pooled and used for this experiment.
(TIF)

S5 Fig Simulated noise behavior of an ensemble of cells having two subpopulations. Both the subpopulations have lognormal distribution. One subpopulation has lower mean and variance than the other, and is called “Low cells”. It is equivalent to CR-1 negative population in our experiments. Mean and variance of the other subpopulation (called High cells) is higher and equivalent to CR-1 positive subpopulation in our experiment. The size of this subpopulation was varied from 0 to 100% of the whole population. Similar to our experimental observation, the mean and variance of this subpopulation were increased with increase in its size. We have simulated 20000 cells in one run with a particular set of parameter values. Each run was repeated 1000 times and the average result is shown here. For Low cells: μ = 5 and σ^2^ = 50. For High cells: μ varied from 10 to 100 and γ = σ^2^/μ = 10, 100, and 1000. Simulations were performed using MATLAB. a) Shows change in CV of the whole population with increase in percentage of High cells. b) Shows relation between mean and CV, as the percentage of High cells increases.
(TIF)

S6 Fig Dot-plot to show absence of any correlation between cells size and readings for CR1-PE. Data of a typical experiment with different treatment group is shown here. The straight line in each plot was obtained by linear regression. R^2^: correlation coefficient.
(TIF)

S1 Table Primers for different genes. (DOC)

S2 Table Antibodies used for Western Blots. (DOC)
