# Supplementary figures and images for: Autoregulation and Heterogeneity in Expression of Human Cripto-1
Source: PLoS One. 2015 Feb 6;10(2):e0116748. doi: 10.1371/journal.pone.0116748 (PMC4319928; doi:10.1371/journal.pone.0116748)

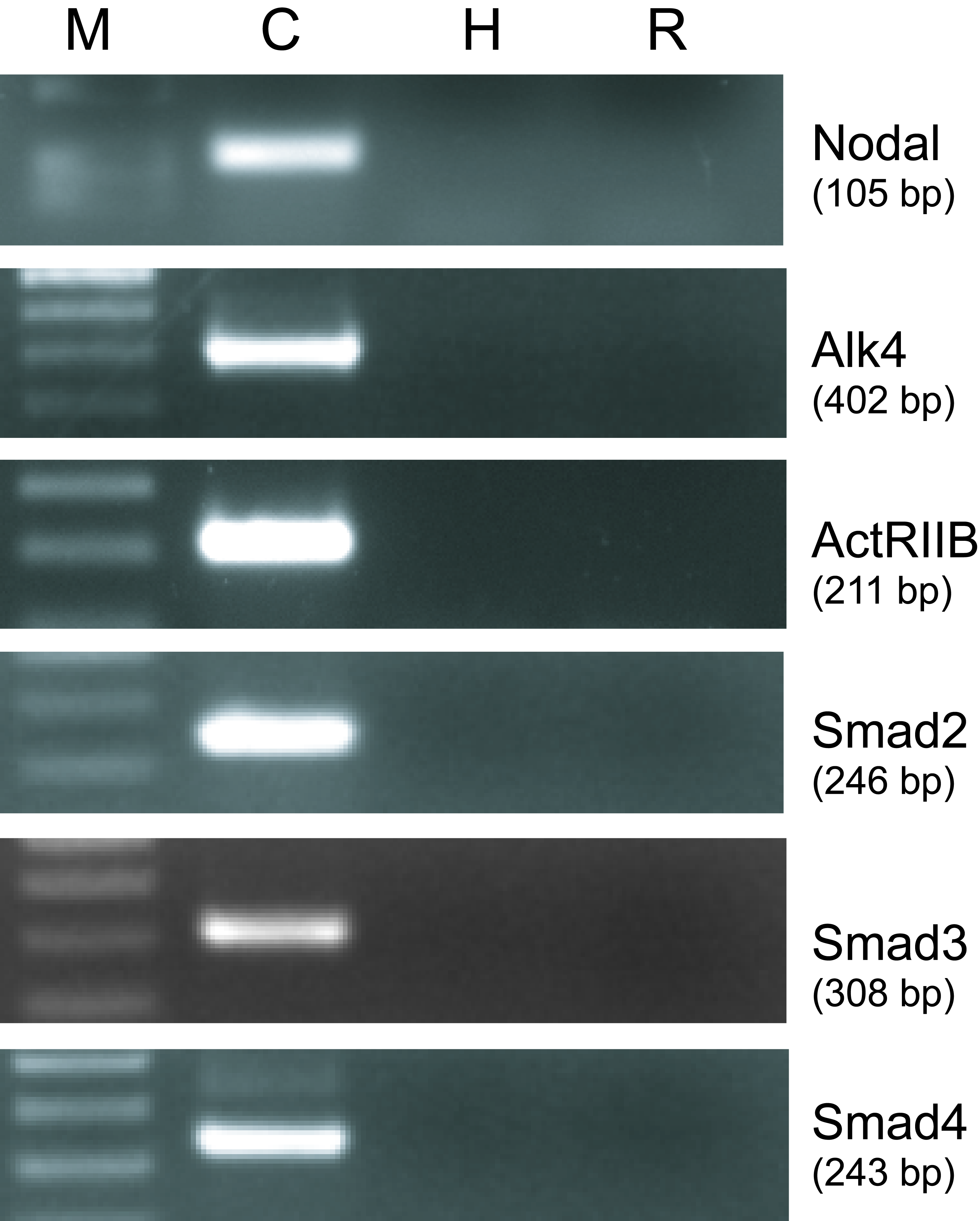

Supplement: S1 Fig — Reverse transcription followed by PCR was used to detect expression of different pathway molecules in U-87 MG cells. C: cDNA, R: RNA, H: water, M: DNA marker. (TIF) [file pone.0116748.s001.tif]

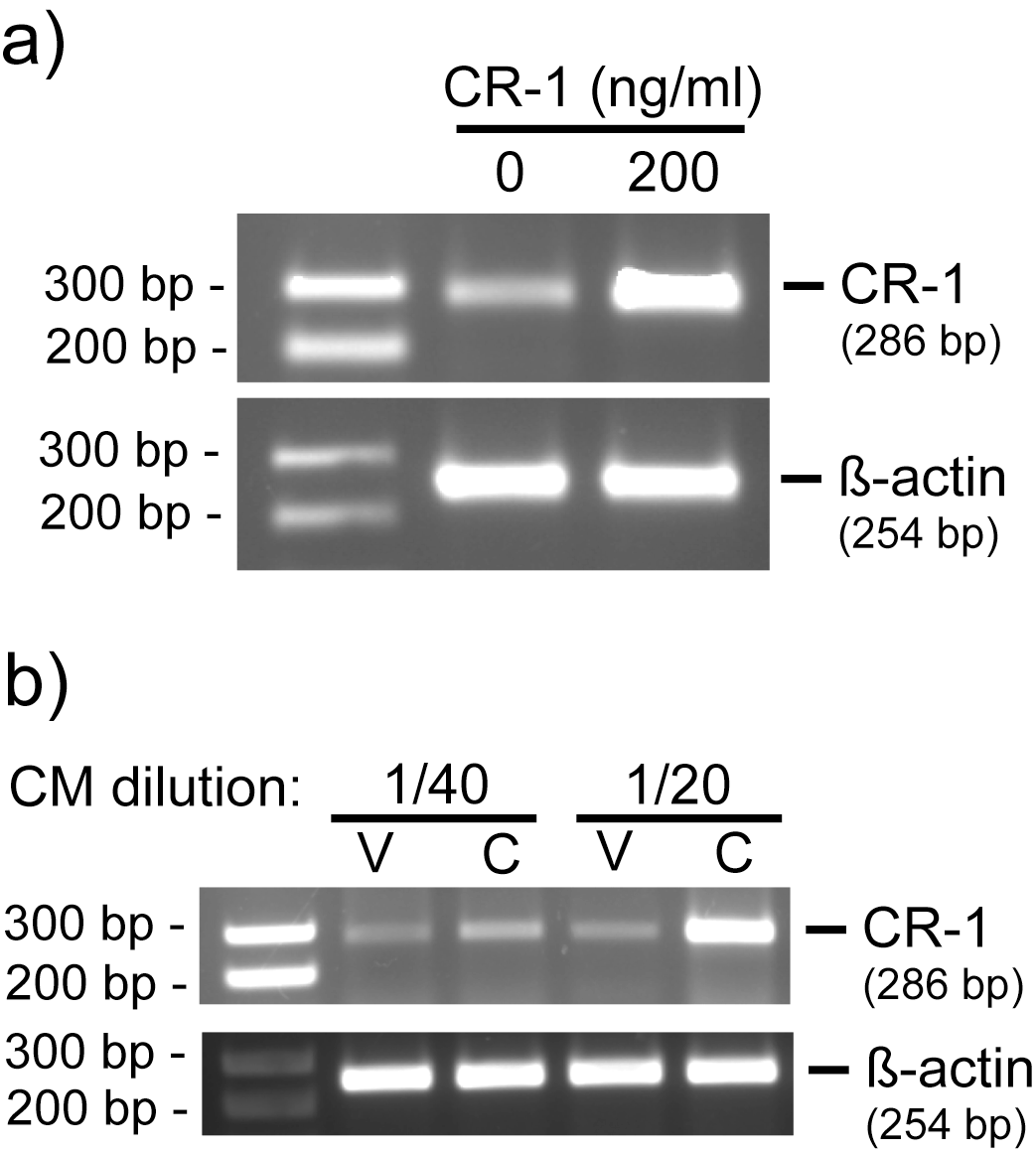

Supplement: S2 Fig — a) U-87 MG cells were treated with recombinant CR-1 (R&D systems) expressed using an insect expression system. b) U-87 MG cells were treated with two dilutions (1:40 and 1:20) of conditioned media of MCF-7 cells overexpressing soluble CR-1 (C) or having transfected with the vector only (V). In both the experiments cells were treated for 24 hr. (TIF) [file pone.0116748.s002.tif]

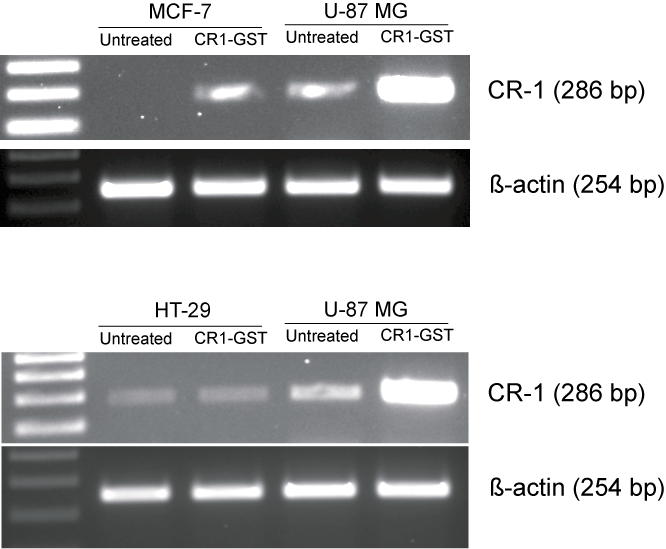

Supplement: S3 Fig — MCF-7, HT-29 and U-87 MG cells were treated with recombinant CR-1 for 24 hr and expression of CR-1 was checked by RT-PCR. (TIF) [file pone.0116748.s003.tif]

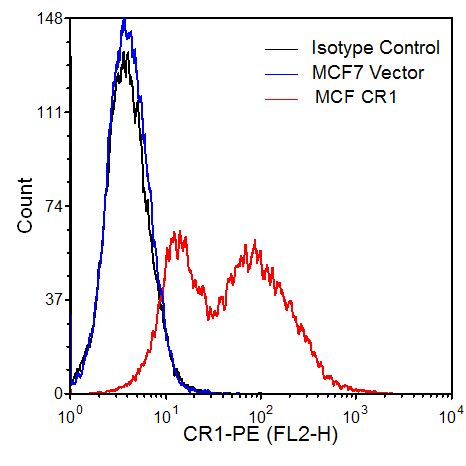

Supplement: S4 Fig — MCF CR1: MCF-7 cells overexpressing full-length CR-1 and stained with the anti-CR-1 antibody PE conjugate; MCF7 vector: MCF-7 cells transfected with empty vector and stained with the anti-CR-1 antibody PE conjugate; Isotype control: MCF-7 cells overexpressing full-length CR-1 and stained with isotype control antibody. All cells were stably transfected. MCF-7 cells overexpressing CR-1 were not monoclonal. Rather, transfected clones selected for drug resistance were pooled and used for this experiment. (TIF) [file pone.0116748.s004.tif]

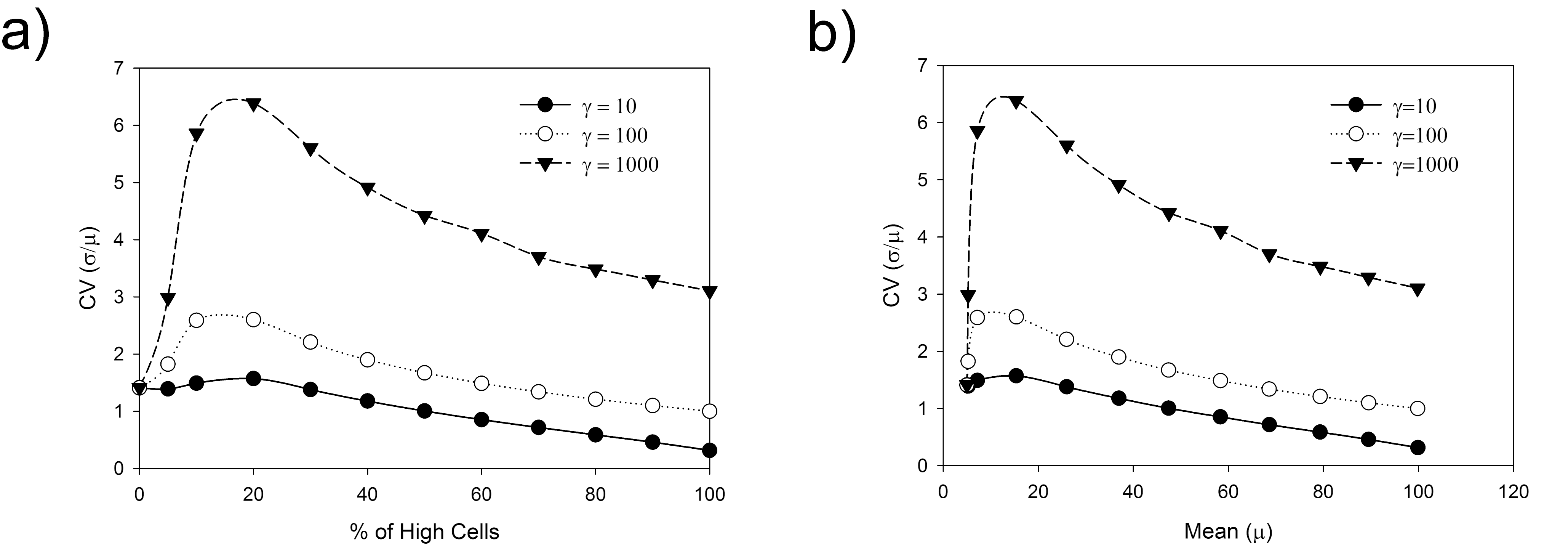

Supplement: S5 Fig — Both the subpopulations have lognormal distribution. One subpopulation has lower mean and variance than the other, and is called “Low cells”. It is equivalent to CR-1 negative population in our experiments. Mean and variance of the other subpopulation (called High cells) is higher and equivalent to CR-1 positive subpopulation in our experiment. The size of this subpopulation was varied from 0 to 100% of the whole population. Similar to our experimental observation, the mean and variance of this subpopulation were increased with increase in its size. We have simulated 20000 cells in one run with a particular set of parameter values. Each run was repeated 1000 times and the average result is shown here. For Low cells: μ = 5 and σ2 = 50. For High cells: μ varied from 10 to 100 and γ = σ2/μ = 10, 100, and 1000. Simulations were performed using MATLAB. a) Shows change in CV of the whole population with increase in percentage of High cells. b) Shows relation between mean and CV, as the percentage of High cells increases. (TIF) [file pone.0116748.s005.tif]

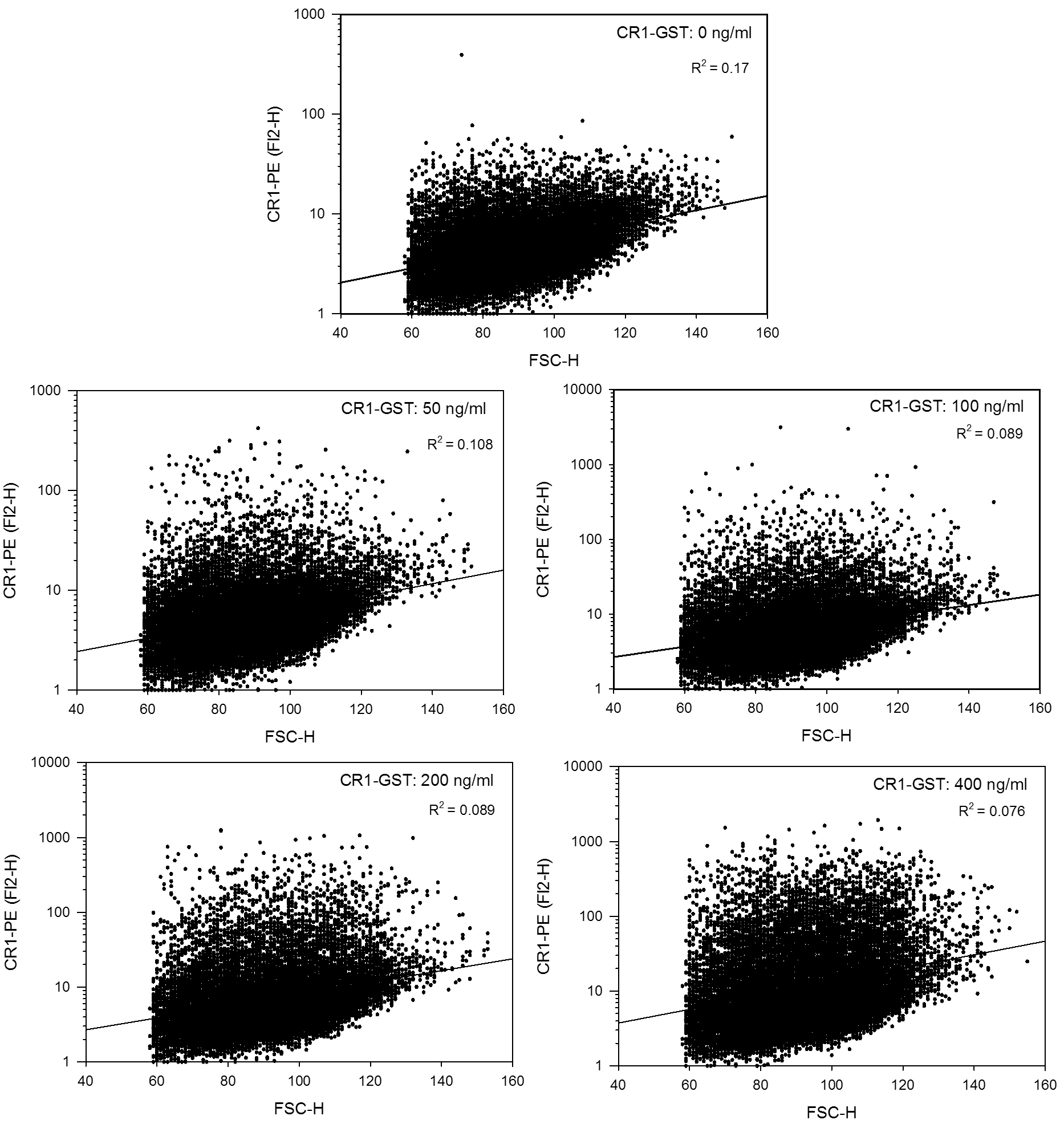

Supplement: S6 Fig — Data of a typical experiment with different treatment group is shown here. The straight line in each plot was obtained by linear regression. R2: correlation coefficient. (TIF) [file pone.0116748.s006.tif]
