# Supplementary material for: Autoregulation and Heterogeneity in Expression of Human Cripto-1
Source: PLoS One. 2015 Feb 6;10(2):e0116748. doi: 10.1371/journal.pone.0116748 (PMC4319928; doi:10.1371/journal.pone.0116748)
Supplement: S1 Table — (DOC) [file pone.0116748.s007.doc]

**Table S**1: Primers for different genes

| **Target** | **Primers** | **Sequences (5’-3’)** | **References** |
| --- | --- | --- | --- |
| Nodal | Forward | CCCAAGCAGTACAACGCCTA | Designed by us |
|  | Reverse | ACGTTTCAGCAGACTCTGGAT |  |
| Alk4 | Forward | CTCCAAAGACAAGACGCTCC | Designed by us |
|  | Reverse | CCAGCTTAATCATCCCCTCA |  |
| ActRIIB | Forward | CACAGCCGTGCTGGCTGACT | Designed by us |
|  | Reverse | TGCAGCCTTGCAGCGAGACA |  |
| Smad2 | Forward | AAGAAGTCAGCTGGTGGGT | [1] |
|  | Reverse | GCCTGTTGTATCCCACTGA |  |
| Smad3 | Forward | CAGAACGTCAACACCAAGT | [2] |
|  | Reverse | ATGGAATGGCTGTAGTCGT |  |
| Smad4 | Forward | CCAGGATCAGTAGGTGGAAT | [1] |
|  | Reverse | GTCTAAAGGTTGTGGGTCTG |  |
| CR-1 | Forward | GATACAGCACAGTAAGGAGC | [3] |
|  | Reverse | TAGTTCTGGAGTCCTGGAAG |  |
| MDR-1 | Forward | GCCTGGCAGCTGGAAGACAAATACACAAAATT | [4] |
|  | Reverse | CAGACAGCAGCTGACAGTCCAAGAACAGGACT |  |
| Oct4 | Forward | GAGAACCGAGTGAGAGGCAACC | Designed by us |
|  | Reverse | CATAGTCGCTGCTTGATCGCTTG |  |
| Nanog | Forward | AATACCTCAGCCTCCAGCAGATG | [5] |
|  | Reverse | TGCGTCACACCATTGCTATTCTTC |  |
| Sox2 | Forward | GCTCGCAGACCTACATGAAC | [6] |
|  | Reverse | GGGAGGAAGAGGTAACCACA |  |
| CD133 | Forward | ATGACAAGCCCATCACAACA | [7] |
|  | Reverse | AGCACTACCCAGAGACCAATG |  |
| β-actin | Forward | CTGTCTGGCGGCACCACCAT | [8] |
|  | Reverse | GCAACTAAGTCATAGTCCGC |  |
| Cyclophilin | Forward | ACACGCCATAATGGCACTGG | [9] |
|  | Reverse | ATTTGCCATGGACAAGATGCC |  |

**References:**

1. Xu G, Chakraborty C, Lala PK (2001) Expression of TGF-beta signaling genes in the normal, premalignant, and malignant human trophoblast: loss of smad3 in choriocarcinoma cells. Biochem Biophys Res Commun 287: 47-55.

2. Xu G, Chakraborty C, Lala PK (2003) Reconstitution of Smad3 restores TGF-beta response of tissue inhibitor of metalloprotease-1 upregulation in human choriocarcinoma cells. Biochem Biophys Res Commun 300: 383-390.

3. Wu Z, Li G, Wu L, Weng D, Li X, et al. (2009) Cripto-1 overexpression is involved in the tumorigenesis of nasopharyngeal carcinoma. BMC Cancer 9: 315.

4. Knutsen T, Mickley LA, Ried T, Green ED, du Manoir S, et al. (1998) Cytogenetic and molecular characterization of random chromosomal rearrangements activating the drug resistance gene, MDR1/P-glycoprotein, in drug-selected cell lines and patients with drug refractory ALL. Genes Chromosomes Cancer 23: 44-54.

5. Willems E, Mateizel I, Kemp C, Cauffman G, Sermon K, et al. (2006) Selection of reference genes in mouse embryos and in differentiating human and mouse ES cells. Int J Dev Biol 50: 627-635.

6. Ferletta M, Caglayan D, Mokvist L, Jiang Y, Kastemar M, et al. (2011) Forced expression of Sox21 inhibits Sox2 and induces apoptosis in human glioma cells. Int J Cancer 129: 45-60.

7. Yin B, Zeng Y, Liu G, Wang X, Wang P, et al. (2014) MAGE-A3 is highly expressed in a cancer stem cell-like side population of bladder cancer cells. Int J Clin Exp Pathol 7: 2934-2941.

8. Mitani Y, Rao PH, Futreal PA, Roberts DB, Stephens PJ, et al. (2011) Novel chromosomal rearrangements and break points at the t(6;9) in salivary adenoid cystic carcinoma: association with MYB-NFIB chimeric fusion, MYB expression, and clinical outcome. Clin Cancer Res 17: 7003-7014.

9. Aloisi AM, Ceccarelli I, Fiorenzani P, Maddalena M, Rossi A, et al. (2010) Aromatase and 5-alpha reductase gene expression: modulation by pain and morphine treatment in male rats. Mol Pain 6: 69.
