# Supplementary material for: Autoregulation and Heterogeneity in Expression of Human Cripto-1
Source: PLoS One. 2015 Feb 6;10(2):e0116748. doi: 10.1371/journal.pone.0116748 (PMC4319928; doi:10.1371/journal.pone.0116748)
Supplement: S2 Table — (DOC) [file pone.0116748.s008.doc]

Table S2: Antibodies used for Western Blots

|  | Antibody | Dilution used |
| --- | --- | --- |
| Primary antibody | Goat anti-human CR-1  (R&D systems, Cat. No. AF145) | 1:500 |
|  | Rabbit anti-human β-actin  (Cell Signaling Technology, Cat. No. 4970) | 1:3000 |
|  | Rabbit anti-human phospho-SMAD2 (Ser465/467)  (Cell Signaling Technology, Cat. No. 3108) | 1:1000 |
|  | Rabbit anti-human SMAD2  (Cell Signaling Technology, Cat. No. 3102) | 1:3000 |
|  | Rabbit anti- human COX IV  (Cell Signaling Technology, Cat. No. 4850) | 1:3000 |
| Secondary Antibody | Rabbit anti Goat-HRP  (Bangalore Genei, Cat. No. 105500) | 1:3000 |
|  | Goat anti Rabbit-HRP  (Cell Signaling, U.S.A, Cat. No. 7074) | 1:6000 |
